# Development of a multi‐recombinase polymerase amplification assay for rapid identification of COVID‐19, influenza A and B

**DOI:** 10.1002/jmv.28139

**Published:** 2022-09-20

**Authors:** Li‐Guo Liang, Miao‐jin Zhu, Rui He, Dan‐Rong Shi, Rui Luo, Jia Ji, Lin‐Fang Cheng, Xiang‐Yun Lu, Wei Lu, Fu‐Ming Liu, Zhi‐Gang Wu, Nan‐Ping Wu, Hang Chen, Zhe Chen, Hang‐Ping Yao

**Affiliations:** ^1^ State Key Laboratory for Diagnosis and Treatment of Infectious Diseases The First Affiliated Hospital, Zhejiang University School of Medicine Hangzhou China; ^2^ National Clinical Research Center for Infectious Diseases, The First Affiliated Hospital Zhejiang University School of Medicine Hangzhou China; ^3^ Center for Clinical Laboratory The First Affiliated Hospital of Zhejiang Chinese Medical University Hangzhou China; ^4^ Zhejiang Center for Medical Device Evaluation Hangzhou Zhejiang China; ^5^ The Key Laboratory of Biomedical Engineering of Ministry of Education, College of Biomedical Engineering and Instrument Science Zhejiang University Hangzhou China

**Keywords:** COVID‐19, influenza, laboratory diagnosis, RPA, SARS‐CoV‐2

## Abstract

The coronavirus disease 2019 (COVID‐19) pandemic caused extensive loss of life worldwide. Further, the COVID‐19 and influenza mix‐infection had caused great distress to the diagnosis of the disease. To control illness progression and limit viral spread within the population, a real‐time reverse‐transcription PCR (RT‐PCR) assay for early diagnosis of COVID‐19 was developed, but detection was time‐consuming (4–6 h). To improve the diagnosis of COVID‐19 and influenza, we herein developed a recombinase polymerase amplification (RPA) method for simple and rapid amplification of Severe Acute Respiratory Syndrome Coronavirus 2 (SARS‐CoV‐2), the causative agent of COVID‐19 and Influenza A (H1N1, H3N2) and B (influenza B). Genes encoding the matrix protein (M) for H1N1, and the hemagglutinin (HA) for H3N2, and the polymerase A (PA) for Influenza B, and the nucleocapsid protein (N), the RNA‐dependent‐RNA polymerase (RdRP) in the open reading frame 1ab (ORF1ab) region, and the envelope protein (E) for SARS‐CoV‐2 were selected, and specific primers were designed. We validated our method using SARS‐CoV‐2, H1N1, H3N2 and influenza B plasmid standards and RNA samples extracted from COVID‐19 and Influenza A/B (RT‐PCR‐verified) positive patients. The method could detect SARS‐CoV‐2 plasmid standard DNA quantitatively between 10^2^ and 10^5^ copies/ml with a log linearity of 0.99 in 22 min. And this method also be very effective in simultaneous detection of H1N1, H3N2 and influenza B. Clinical validation of 100 cases revealed a sensitivity of 100% for differentiating COVID‐19 patients from healthy controls when the specificity was set at 90%. These results demonstrate that this nucleic acid testing method is advantageous compared with traditional PCR and other isothermal nucleic acid amplification methods in terms of time and portability. This method could potentially be used for detection of SARS‐CoV‐2, H1N1, H3N2 and influenza B, and adapted for point‐of‐care (POC) detection of a broad range of infectious pathogens in resource‐limited settings.

**Abbreviations**
COVID‐19: coronavirus disease 2019
LAMP: loop‐mediated isothermal amplification
POC: point‐of‐care
RPA: recombinase polymerase amplification
RT‐PCR: reverse‐transcription PCR

## 1 INTRODUCTION

Coronavirus disease 2019 (COVID‐19), caused by severe acute respiratory syndrome coronavirus 2 (SARS‐CoV‐2), was first reported in Wuhan, China, in late 2019.^1^ The main symptoms include fever, shortness of breath, asthenia, and coughing, accompanied by nasal congestion, a runny nose, diarrhea and other upper respiratory tract and digestive tract symptoms in some patients.^2^, ^3^, ^4^ These symptoms resemble those of patients with influenza or the common cold, resulting in misdiagnosis initially. COVID‐19 rapidly spread worldwide, with 602 136492 confirmed cases reported by August 22, 2022. Since the end of 2019, COVID‐19 has caused extensive loss of life and severe economic losses worldwide,^5^, ^6^ killing more than 6 455 497 people by August 22, 2022, with a mortality rate of 2%.^7^ In the past two years, the mutation of the virus has accelerated its global epidemic, especially the Delta and Omicron variants, and the number of infections around the world is still increasing dramatically. Although numerous gene amplification assays have been developed for virus detection, they are time‐consuming and often suffer from poor sensitivity.^8^, ^9^, ^10^, ^11^ It is therefore urgent to develop an accurate, rapid point‐of‐care (POC) diagnosis method that can effectively identify infections and carriers to prevent the virus spreading.

To control illness progression and limit viral spread within the population, various methods (computed tomography [CT] scan, syndromic testing, nucleic acid testing and antibody testing) have been developed for early detection.^9^, ^11^, ^12^, ^13^, ^14^ Real‐time reverse‐transcription fluorescent polymerase chain reaction (RT‐PCR) of viral RNA from upper respiratory tract samples (i.e., nasopharyngeal swabs, nasal aspirates, and nasopharyngeal washes) is considered the gold‐standard method for clinical diagnosis of COVID‐19.^8^, ^10^, ^12^, ^15^ Genes encoding the nucleocapsid protein (N), the RNA‐dependent‐RNA polymerase (RdRP) in the open reading frame 1ab (ORF1ab) region, and the envelope protein (E) have been used to design primers and probes to detect SARS‐CoV‐2. For example, Corman and colleagues^15^ aligned and analyzed several SARS‐related viral genome sequences to design a set of primers and probes, and developed an RT‐PCR method to detect SARS‐CoV‐2. In their study, both the E and RdRP genes achieved high sensitivity (limit of detection of 3.9 copies and 3.6 copies per reaction, respectively) for detection, whereas the N gene yielded poorer sensitivity (8.3 copies per reaction).^7^ However, the RT‐PCR method is time‐consuming (4–6 h), labor‐intensive, and instrument‐dependent. Furthermore, a major challenge for RT‐PCR is the difficulty in optimizing the amplification and reverse transcription steps because they occur simultaneously, which leads to lower target amplicon generation.

Loop‐mediated isothermal amplification (LAMP) is a method that can amplify nucleic acids with high specificity, sensitivity, and rapidity at 60°C–65°C, that does not require expensive reagents or special instruments such as a thermal cycler. In 2003, this method was used for SARS coronavirus detection.^16^ Several academic laboratories have developed and clinically tested RT‐LAMP tests for SARS‐CoV‐2 detection. Yu and colleagues developed an RT‐LAMP diagnostic platform for COVID‐19,^13^ but the sensitivity was low (limit of detection of 60 copies/μL), and the target region was only a fragment of ORF1ab, which may lead to failed diagnosis. The same group also reported a rapid and visual detection method for SARS‐CoV‐2 based on RT‐LAMP^14^ using primers specific for the spike protein (S) and ORF1ab genes of SARS‐CoV‐2, and detection could be achieved within approximately 30 min. However, this assay also suffered from low sensitivity for the S gene (2 × 10^2^ copies per reaction). In addition to isothermal amplification, there are other nucleic acid tests that can be used for SARS‐CoV‐2 detection. For example, SHERLOCK technology is a detection strategy that uses Cas13a for RNA sensing.^17^, ^18^, ^19^ Zhang and colleagues reported a SHERLOCK protocol for detecting SARS‐CoV‐2^20^ involving three steps that can be completed in 1 h, starting from nucleic acid extraction, as used for RT‐PCR tests for S and ORF1ab genes. However, the limit of detection ranged between 10 and 100 copies/μl of SARS‐CoV‐2 RNA sequence. Clearly, more accurate, and rapid diagnostic methods are needed for diagnosis of COVID‐19 during the early stages of screening.

RPA is an isothermal amplification technique for the specific, rapid, and cost‐effective detection of pathogens. Due to its low operation temperature (37°C–42°C) and commercial availability of freeze‐dried reagents, it has been applied outside laboratory settings in remote areas.^21^, ^22^, ^23^, ^24^ According to previous study,^25^ two recombinase‐based isothermal techniques, reverse transcription recombinase polymerase amplification (RT‐RPA) and reverse transcription recombinase‐aided amplification (RT‐RAA), were evaluated for the detection of SARS‐CoV‐2 in clinical samples (e.g., 176 cases). The results showed that sensitivity of RT‐RPA and RT‐RAA was only 85.53% and 76.32%, respectively. In another study, one‐tube SARS‐CoV‐2 detection platform based on RT‐RPA and CRISPR/Cas12a was reported.^26^ This method has high sensitivity (a low detection limit of 2.5 copies/µl input (RNA standard) and 1 copy/µl input (pseudovirus)), but the detection time is more than 50 min. An RPA and AuNP‐based colorimetric assay were also be used for SARS‐CoV‑2 detection.^27^ In their study, they can specifically target an ORF1ab and N regions of the SARS‐CoV‐2 genome, and bring the sensitivity of the method to one copy of viral genome sequence per test, however, there was no actual test validation with clinical samples.

What's more serious is that the mixed infection of SARS‐CoV‐2 and influenza A or B has caused great trouble to clinical diagnosis and treatment. The susceptibility of COVID‐19 in influenza‐infected people is enhanced, and the condition of mixed infection of COVID‐19 is aggravated, which is easy to develop into severe pneumonia in the present work.^28^, ^29^ We developed a more convenient and faster method for detecting SARS‐CoV‐2, H1N1, H3N2, and influenza B based on recombinase polymerase amplification (RPA) technology. In our study, N, E and ORF1ab genes of SARS‐CoV‐2 and genes encoding the matrix protein (M) for H1N1, and the hemagglutinin (HA) for H3N2, and the polymerase A (PA) for Influenza B were selected and specific primers were designed. We validated our method using SARS‐CoV‐2 pseudovirus standards and RNA samples extracted from COVID‐19, influenza (RT‐PCR‐verified) positive patients. Our method could detect SARS‐CoV‐2 using the RPA method with a sensitivity of 100% at a specificity of 96.67%, 96.67%, and 100% for N, ORF1ab, and E gene, respectively. Also, the sensitivity of 100% at a specificity of 100%, 100%, and 90% for M, HA, and PA gene, respectively. Our RPA method could provide a point‐of‐care (POC) detection assay for rapid identification of COVID‐19 and influenza patients.

## 2 MATERIALS AND METHODS

### 2.1 Materials

SARS‐CoV‐2 plasmid DNA were obtained from Yeasen Biotech Co., Ltd. H Cov‐229E plasmid DNA, H Cov‐MERS plasmid DNA, H Cov‐SARS plasmid DNA and H Cov‐OC43 plasmid DNA were obtained from Fubio Biological Technology Co., Ltd. All the plasmid DNA contained the target gene fragment and quantified. Influenza A virus H1N1, H3N2, H5N1, H7N9 and Influenza B and 14 strains of bacteria have been identified from our laboratory. RT‐exo kits (Reverse transcription‐enhanced recombinant amplification Kit, RT‐ERA) for amplification were purchased from Suzhou Xianda Gene Technology Co., Ltd. MolPure® Magnetic Swab Viral DNA/RNA Kit for RNA extraction was purchased from Yeasen Biotech Co., Ltd (Shanghai, China). Primers (e.g., SARS‐CoV‐2 forward and reverse primers, FAM‐labeled exo probes) and nuclease‐free H_2_O were obtained from Sangon Biotech Co., Ltd. RT‐PCR kits were purchased from Shanghai ZJ Bio‐Tech Co., Ltd.

### 2.2 Clinical RNA samples for COVID‐19 and influenza patients

RNA samples were obtained from 70 confirmed cases diagnosed with COVID‐19 and 50 confirmed cases diagnosed with influenza from The First Affiliated Hospital, School of Medicine, Zhejiang University. These samples were used for developing our method. The study was approved by the Clinical Research Ethics Committee of the First Affiliated Hospital, School of Medicine, Zhejiang University (Approval No. IIT20200233A).

### 2.3 Primer design

Specific RPA primers for SARS‐CoV‐2, H1N1, H3N2 and influenza B were designed based on the E, N and ORF1ab sequences (NCBI GenBank Accession No. NC_045512.2) and the M, HA (NCBI GenBank Accession No. NC_026431.1and NC_026433.1), and PA sequence (NCBI GenBank Accession No. NC_002206.1) using the primer design software (Primer Premier 5) and the specific RPA probe primers follows the design principles of RPA probes and were designed by the author of the paper. The three groups of primers are shown in Table 1. All primers were synthesized commercially (Sangon Biotech Co., Ltd.).

**Table 1:** Primers for amplifying N, E, ORF1ab, M, HA, and PA genes by RPA assay

Target gene	Labeled	Sequence (5′−3′)
	F1	AACTAATCAGACAAGGAACTGATTACAAAC
N gene of SARS‐CoV‐2	R1	CTTATTCAGCAAAATGACTTGATCTTTGAA
	P1	GCTTCAGCGTTCTTCGGAATGTCGCGCAT/i6FAMdT/GG/idSp/A/iBHQ1dT/GGAAGTCACACCTTCGGG/iSpC3/
	F2	GAAGCGACAACAATTAGTTTTTAGGAATTTA
E gene of SARS‐CoV‐2	R2	CTAAAGGATTTTGTGACTTAAAAGGTAAGTAT
	P2	GCGGTATGTGGAAAGGTTATGGCTGTAGT/i6FAMdT/G/idSp/GA/iBHQ1dT/CAACTCCGCGAAC
	F3	TAGTTAATAGCGTACTTCTTTTTCTTGCTT
ORF1ab gene of SARS‐CoV‐2	R3	GAATTCAGATTTTTAACACGAGAGTAAACG
	P3	CACTAGCCATCCTTACTGCGCTTCGATTGTG/i6FAMdT/G/idSp/G/iBHQ1dT/ACTGCTGCAATATTGTT/iSpC3/
	F4	CTGACTAAGGGAATTTTAGGATTTGTGTTC
M gene of H1N1	R4	GTATAGTTTAACTGCTCTATCCATGTTGTT
	P4	CCCTAAATGGGAATGGGGACCCGAACAACA[i6ROXdT] G[iBHQ1dT] A[idSp]AGAGCAGTTAAACTA [iSpC3]
HA gene of H3N2	F5	GACACTAAAATAGATCTCTGGTCATACAAC
	R5	CATCTCTGTATACATCATGGTCATAAGTTC
	P5	GCCCTGGAGAACCAACATACAATTGATCTAAC[i6ROXdT] G[idSp]C[iBHQ1dT] CAGAAATGAACA [iSpC3]
	F6	TATTAAATGAAAGCAATGCTAGTATGGGAA
PA gene of influenza B	R6	ATTTCATTTGGATTTTGTTTGTACCATTCA
	P6	CACGGATGTTGTAACAGTTGTGACTT[i6CY3dT]CG [idSp]G[iBHQ1dT] TTAGTAGTACAGATCCTAG[iSpC3]

### 2.4 Preparation of SARS‐CoV‐2, H1N1, H3N2, and influenza B standards

The concentration of plasmid DNA containing partial fragments of the ORF1ab, N and E genes of SARS‐CoV‐2 and the M, HA, and PA genes of H1N1, H3N2 and influenza B was quantified, and samples were serially 10‐fold diluted from 1 × 10^6^ to 1 × 10^0^copies/ml as standard templates.

### 2.5 RPA and real‐time PCR assays

Each 50 μl RPA reaction consisted of 2.1 μl of 10 μM forward primer, 2.1 μl of 10 μM S reverse primer, 0.6 μl of 10 μM exo probe for SARS‐CoV‐2, H1N1, H3N2, and influenza B, respectively (Tables 1), 29.5 μl of rehydration buffer, 3.2 μl of nuclease‐free water, 2.5 μl of magnesium acetate, and 10 μl of plasmid (10‐fold diluted from 1–10^6^ copies/ml) or RNA samples. And, all liquid mixtures were added separately to reaction tubes containing lyophilized enzymes (kit). Negative controls contained nuclease‐free water instead of plasmid DNA. The mixture was transferred to a real‐time PCR machine and the fluorescent intensity was measured. Samples were incubated at 39°C for 30 min, and fluorescence intensity was measured every 30 s during the amplification process. Quantitation cycle (Cq) values were measured automatically by the thermocycler when the fluorescence exceeded the background signal, and a standard curve was constructed to analyse the correlation between DNA concentrations and Cq values.

Real‐time PCR amplification was performed in a 50 μl reaction mixture containing 10 μl of Rnase‐free water, 25 μl of Premix Ex Taq (Probe qPCR), 10 μl of DNA template, 1 μl (10 μmol) of probe primer, and 2 μl (10 μmol) of forward and reverse primers. The thermal cycling protocol included an initial denaturation step at 95°C for 30 s, followed by 40 cycles of denaturation at 95°C for 5 s and extension at 60°C for 34 s.

### 2.6 Determination of RPA sensitivity and specificity

To determine the sensitivity of the RPA method, plasmid DNA standards (from 1 copy/ml to 1 × 10^6^ copies/ml) were prepared as templates or detection of the N, E, ORF1ab, M, HA, and PA genes of the plasmid, and RPA assays were performed according to the reaction conditions.

To validate the specificity of the primers, RPA assays were performed using phosphate‐buffered saline (PBS), SARS‐CoV‐2 RNA template (including Delta and Omicron variants), the other viruses (H Cov‐229E, H Cov‐MES, H Cov‐SARS, H Cov‐OC43, Influenza A virus H1N1, H3N2, H5N1, H7N9, and Influenza B) and bacteria.

### 2.7 Clinical validation of the RPA method and statistical analysis

To validate the accuracy of the RPA assay, 70 RNA samples, which were extracted using by RNeasy Mini Kit according to the instruction, from patients diagnosed with COVID‐19 and Influenza A or B (*n* = 55) and normal donors (*n* = 60) were amplified using the RPA method developed herein. All clinical samples were obtained from The First Affiliated Hospital, College of Medicine, Zhejiang University (IRB No. 1019). Box‐Whisker analysis was performed using Origin 8.0 (OriginLab). Receiver operating characteristic (ROC) curves were plotted for assessment of sensitivity and specificity. Two‐sided Student's *t‐*tests were performed using GraphPad Prism 8 (GraphPad Software/https://www.graphpad.com/company/), and *p* < 0.05 was used to indicate significant differences.

### 2.8 Evaluation of the application of the Delta and Omicron strains

To better evaluate whether this method can effectively detect the delta and omicron variants of virus strains. We selected 30 RNA samples of delta virus variant strains (*n* = 18) and pseudovirus fragments of Omicron strain (*n* = 12), and used the detection method established in this study for verification to verify the validity of the method.

## 3 RESULTS

### 3.1 Working principle of the detection method

**Figure 1:**
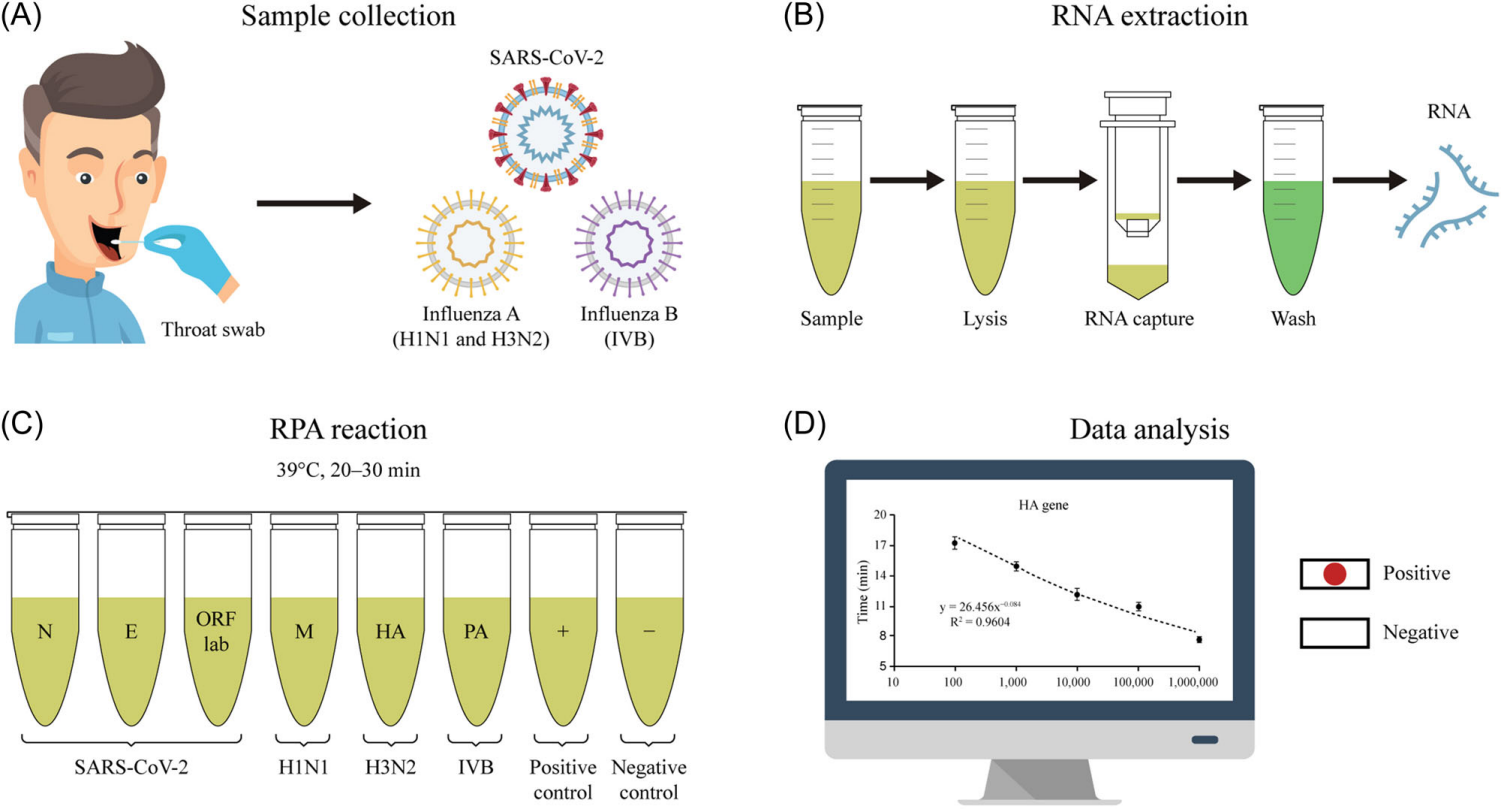
The principle of RPA test for screening of COVID‐19 and influenza patients. (A) Nasopharyngeal swab samples that may contain virus were obtained from patients by medical staff. (B) The viral nucleic acid from the samples was obtained according to the instruction of viral RNA extraction. (C) Following RNA extraction, the RNA samples were screening via an RPA method in Octet PCR tube. (D) The amplification data were sent to a laptop data analysis. COVID‐19, coronavirus disease 2019; PCR, polymerase chain reaction; RPA, recombinase polymerase amplification

Nasopharyngeal swab samples (Figure 1A) that may contain virus were obtained from patients by medical staff. The viral nucleic acid was obtained according to the instruction of MolPure® Magnetic Swab Viral DNA/RNA Kit (Figure 1B). Following RNA extraction, the RNA samples were screening via an RPA method in Octet PCR tube (Figure 1C) and last the amplification data were sent to a laptop data analysis (Figure 1D).

### 3.2 Sensitivity of the RPA assay

**Figure 2:**
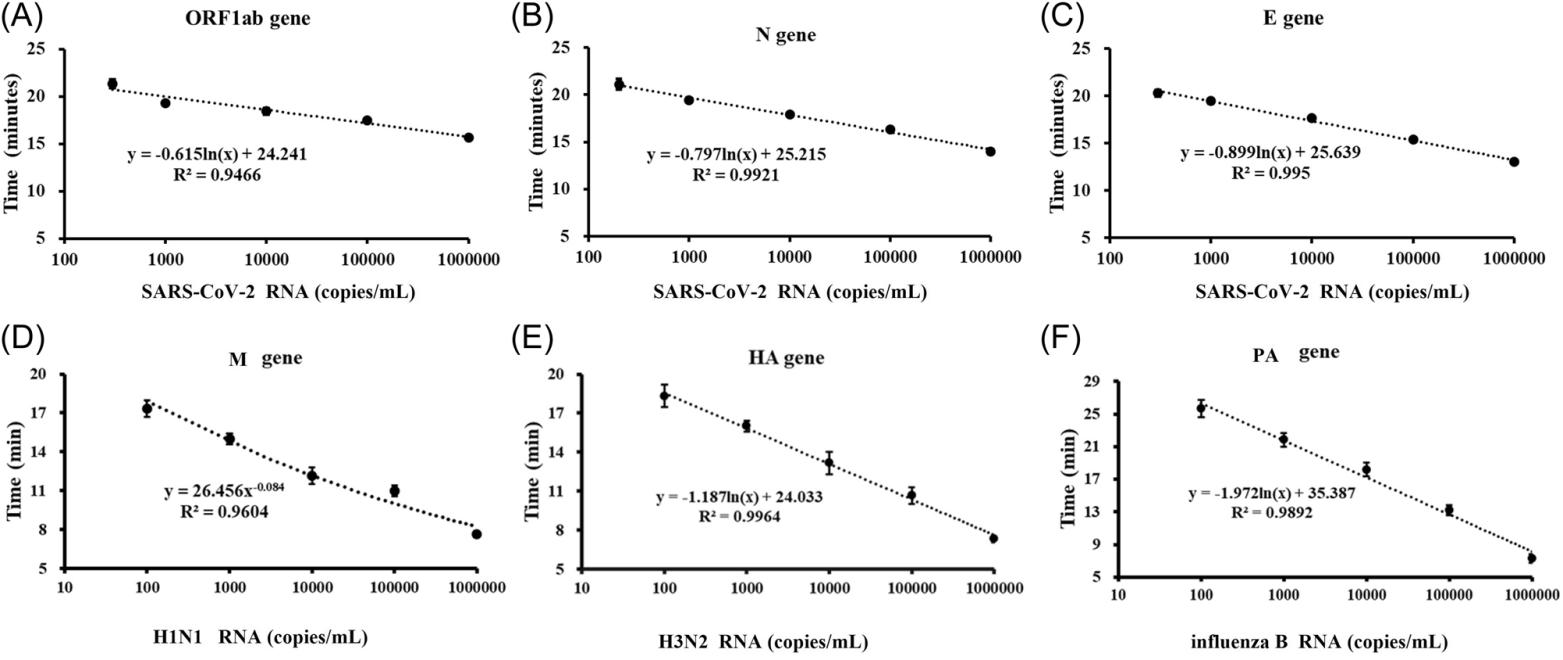
Sensitivity of SARS‐CoV‐2, H1N1, H3N2 and IVB by RPA assay. (A–C) Sensitivity of the RPA assay for ORF1ab, N, and E gene of SARS‐CoV‐2. (D) Sensitivity of the RPA assay for M gene of H1N1. (E) Sensitivity of the RPA assay for HA gene of H3N2. (F) Sensitivity of the RPA assay for PA gene of IVB. RPA, recombinase polymerase amplification; SARS‐CoV‐2, severe acute respiratory syndrome coronavirus 2.

The sensitivity of the RPA assay using primer sets (Table 1) for E, N, ORF1ab, M, HA, and PA genes was evaluated by a quantitative PCR instrument. A series of 10‐fold dilutions of SARS‐CoV‐2, H1N1, H3N2, and influenza B plasmid DNA, ranging from 1 copy per reaction to 10 000 copies per reaction, were analyzed by RPA assay. As shown in Figure 2, the time taken for positive detection ranged from 14.38 min at 10 000 copies per reaction to 20.76 min at 2 copies per reaction using primer set N, whereas for primer set ORF1ab the time taken for positive detection ranged from 15.5 min at 10 000 copies per reaction to 21.7 min at 3 copies per reaction. For primer set E, the detection time ranged from 11.3 min at 10 000 copies per reaction to 19.2 min at 3 copies per reaction. For primer set M, the detection time ranged from 7.7 min at 10 000 copies per reaction to 18.2 min at 1 copy per reaction. For primer set HA, the detection time ranged from 6.3 min at 10 000 copies per reaction to 17.6 min at 1 copy per reaction. For primer set PA, the detection time ranged from 6.6 min at 10 000 copies per reaction to 24.2 min at 1 copy per reaction. Thus, the sensitivity of the assay was 2 copy, 3 copies, and 3 copies per reaction at 39°C within 30 min with primer sets N, ORF1ab, and E, respectively. And the sensitivity of the assay was 1 copy, 1 copy, and 1 copy per reaction at 39°C within 30 min with primer sets M, HA, and PA, respectively A linear correlation between SARS‐CoV‐2 plasmid DNA concentration and time value was observed (Figure 2), with an *R* ^2^ value of 0.99, 0.94, 0.99, 0.96, 0.99, and 0.98 for N, ORF1ab, E, M, HA, and PA, respectively. These results showed that the detection of limit for RPA testing of SARS‐CoV‐2, H1N1, H3N2, and influenza B was 300 copies/ml (due to E gene), 100 copies/ml, 100 copies/ml, and 100 copies/ml, respectively.

### 3.3 Specificity of the RPA assay

To validate the specificity of the RPA approach, PBS, RNA from Influenza and B, and five COVID‐19 negative samples were subjected to RPA analysis as described above. As shown in Table 2, the selected primer sets only amplified the genes of SARS‐CoV‐2, H1N1, H3N2, and IVB, with no cross‐reaction observed for H5N1, H7N9 or B, H Cov‐229E, H Cov‐MES, H Cov‐SARS, H Cov‐OC43, or 14 bacteria or PBS negative controls. These results confirmed that the RPA assay using the optimized primer sets was specific for SARS‐CoV‐2, H1N1, H3N2, and influenza B detection.

**Table 2:** Specificity verification of different samples

Primers species	SARS‐CoV‐2			H1N1 M	H3N2 HA	Influenza B PA
	N	ORF1ab	E			
SARS‐CoV‐2	**+**	**+**	**+**	‐	‐	‐
Delta variants (18 strains)	**+**	**+**	**+**	‐	‐	‐
Omicron variants (12 strains)	**+**	**+**	**+**	‐	‐	‐
H1N1	‐	‐	‐	**+**	‐	‐
H3N2	‐	‐	‐	‐	**+**	‐
H5N1	‐	‐	‐	‐	‐	‐
H7N9	‐	‐	‐	‐	‐	‐
Influenza B	‐	‐	‐	‐	‐	**+**
H Cov‐229E	‐	‐	‐	‐	‐	‐
H Cov‐SARS	‐	‐	‐	‐	‐	‐
H Cov‐MES	‐	‐	‐	‐	‐	‐
H Cov‐OC43	‐	‐	‐	‐	‐	‐
*Streptococcus pneumoniae*	‐	‐	‐	‐	‐	‐
*Haemophilus influenzae*	‐	‐	‐	‐	‐	‐
*Streptococcus haemolyticus*	‐	‐	‐	‐	‐	‐
*Staphylococcus aureus*	‐	‐	‐	‐	‐	‐
*Acinetobacter baumannii*	‐	‐	‐	‐	‐	‐
*Maltophilia*	‐	‐	‐	‐	‐	‐
*Klebsiella aerogenes*	‐	‐	‐	‐	‐	‐
*klebsiella pneumoniae*	‐	‐	‐	‐	‐	‐
*Burkholderia esculenta*	‐	‐	‐	‐	‐	‐
*Pseudomonas aeruginosa*	‐	‐	‐	‐	‐	‐
*Staphylococcus epidermidis*	‐	‐	‐	‐	‐	‐
*Aeromonas hydrophila*	‐	‐	‐	‐	‐	‐
*Aeromonas caviae*	‐	‐	‐	‐	‐	‐
*Moraxella catarrhalis*	‐	‐	‐	‐	‐	‐
PBS	‐	‐	‐	‐	‐	‐

Abbreviation: SARS‐CoV‐2, severe acute respiratory syndrome coronavirus 2.

### 3.4 Strategies for SARS‐CoV‐2 and influenza detection by RPA

In general, laboratory confirmation of positive cases requires that both SRAS‐CoV 2 targets (ORF1ab, N) are positive by real‐time RT‐PCR in the same specimen. If there is a positive test result for a single target, re‐sampling and re‐testing are required. If it is still positive for a single target, it is judged positive. In general, laboratory confirmation of positive cases requires that both novel coronavirus 2 targets (ORF1ab, N) are positive by real‐time RT‐PCR in the same specimen. If there is a positive test result for a single target, re‐sampling and re‐testing are required. If it is still positive for a single target, it is judged positive. To obtain more accurate results, the three genes (N, E and ORF1ab) were combined for detection of SARS‐CoV‐2 (Table 3) and the three genes (M, HA, and PA) were used to detect the H1N1, H3N2 and influenza B, respectively. As shown in Table 3, results of RPA assay were 100% coincidence with qPCR indicated that the established RPA assay can play an important role in the detection of COVID‐19 and influenza A or B.

**Table 3:** Strategies for the RPA assay

Virus	Target genes	PCR‐confirmed	Normal	Results	Diagnosis	Coincidence rate with qPCR
SARS‐CoV‐2	N	70	/	**+**	Positive	100%
	ORF1ab			**+**		
	E			**+**		
SARS‐CoV‐2	N	/	30	**‐**	Negative	100%
	ORF1ab			**‐**		
	E			**‐**		
H1N1	M	20	/	**+**	Positive	100%
	M	/	10	**‐**	Negative	100%
H3N2	HA	20	/	**+**	Positive	100%
	HA	/	10	**‐**	Negative	100%
Influenza B	PA	15	/	**+**	Positive	100%
	PA	/	10	**‐**	Negative	100%

Abbreviations: RPA, recombinase polymerase amplification; SARS‐CoV‐2, severe acute respiratory syndrome coronavirus 2.

### 3.5 Validation of clinical samples

**Figure 3:**
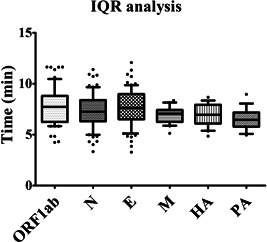
Clinical validation of ORF1ab, E, N, M, HA, PA genes for differentiating COVID‐19 and influenza patients from healthy individuals. (A) 70 RNA samples from COVID‐19 patients were amplified using the RPA method. (B and C) 55 RNA samples from influenza A patients (H1N1, n‐20; H3N2, *n* = 20) were amplified using the RPA method developed herein. (D) 15 RNA samples from influenza B patients were amplified via the RPA method. COVID‐19, coronavirus disease 2019; RPA, recombinase polymerase amplification.

As shown in Figure 3, all 70 COVID‐19 confirmed samples were identified as positive by the RPA assay. The range of time was from 3.39 to 11.17 min, suggesting that the RPA assay could complete detection within 12 min. For the 30 negative samples, all three genes (ORF1ab, N, and E) were negative. Furtherly, RNA samples from influenza patients (*n* = 55; confirmed by RT‐PCR) and normal donors (*n* = 30) were amplified using the RPA method developed herein. Results showed that all the confirmed influenza patients could be detected by our RPA method and there was no amplification for the negative samples. It should be noted that the detection results of Delta variant strains (*n* = 18) and Omicron fragments (*n* = 12) showed that the detection rate of the screening method established in this study was 100% (Table 2).

**Figure 4:**
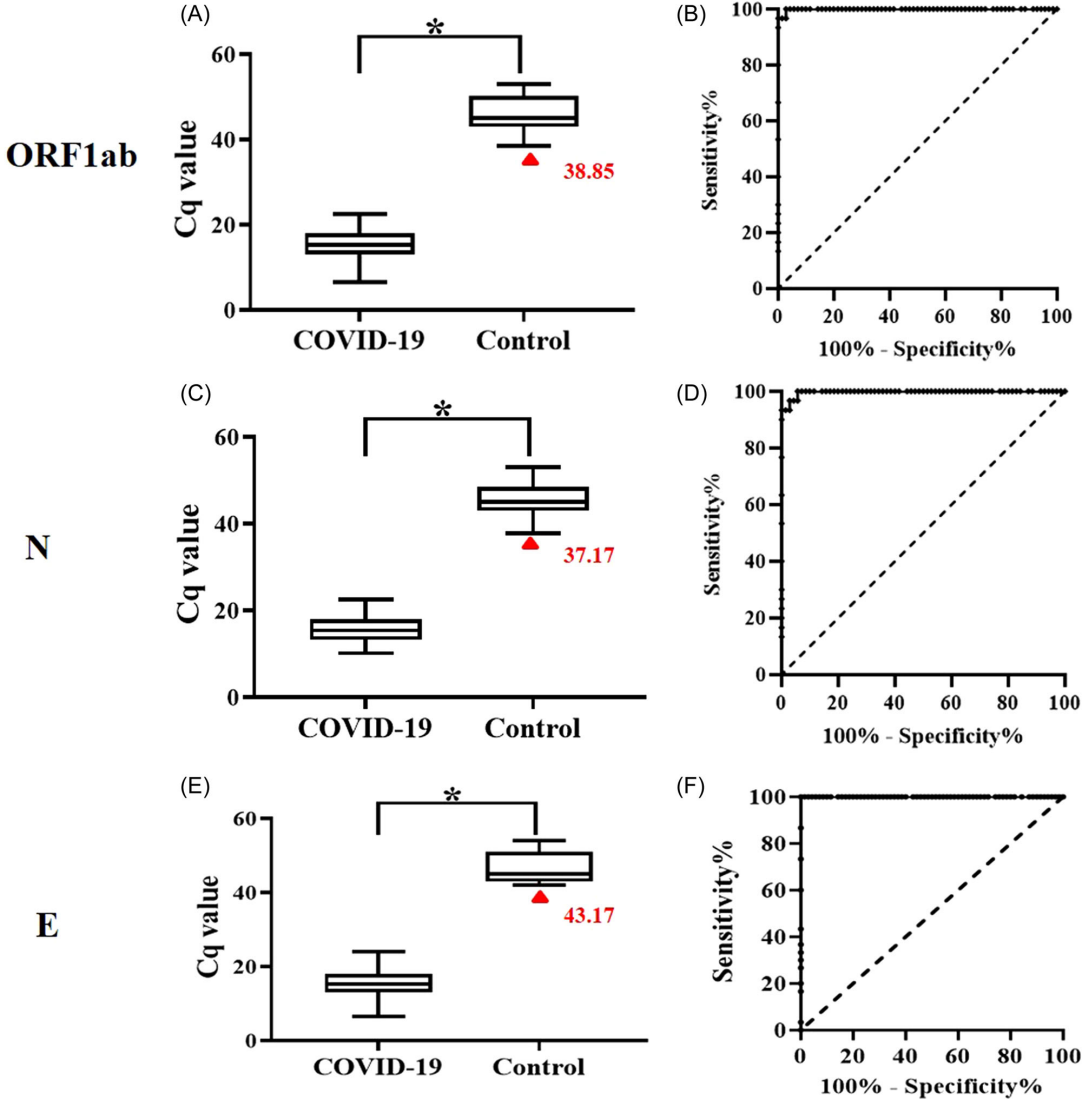
Box‐plot and receiver operating characteristic (ROC) curve analyses. The Cq value of RNA samples from COVID‐19 patients was significantly lower than that from healthy controls (A, C, E). In addition, a ROC curve was plotted for clinical validation, and the RPA assay had a sensitivity of 100% at a specificity of 96.67%, 96.67%, and 100% for N, ORF1ab and E gene, respectively for identifying COVID‐19 patients from healthy controls, with an AUROC value of 0.99 (B, D, F). COVID‐19, coronavirus disease 2019; RPA, recombinase polymerase amplification.

These results indicate that the SARS‐CoV‐2, H1N1, H3N2, and influenza B RNA samples were successfully detected by the developed RPA assay. More importantly, it was found that the Cq value of RNA samples from COVID‐19 patients was significantly lower than that from healthy controls (Figure 4A,C,E). In addition, a ROC curve was plotted for clinical validation, and the RPA assay had a sensitivity of 100% at a specificity of 96.67%, 96.67%, and 100% for N, ORF1ab and E gene, respectively for identifying COVID‐19 patients from healthy controls, with an AUROC value of 0.99 (Figures 4B,D,F). The results demonstrate that the developed RPA assay exhibited good diagnostic performance with clinical samples.

## 4 DISCUSSION

Up to now, the COVID‐19 epidemic is still a global public health event. In this pilot study, we developed a diagnostic method for rapid amplification of SARS‐CoV‐2, H1N1, H3N2, and influenza B nucleic acid samples based on RPA. According to a previous report,^11^, ^14^ one gene alone may be inaccurate due to the rapidly evolving situation. Therefore, in our experiment, we used three target genes to ensure no missed detection and help to increase accuracy especially for the variants (Delta or Omicron). At the same time, to better determine whether the clinical samples are coinfected with COVID‐19 and influenza, we carried out the RPA test design for H1N1, H3N2, and Influenza B (Table 1). It should be noted that for influenza A and influenza B, the probability of rapid mutation is very low, and the detection primers we designed are highly conserved sequences of target genes, and single gene detection is already competent. Compared with influenza A and B viruses' subtype or type, more accurate detection technology is needed for SARS‐CoV‐2 detection, which is also in line with China's dual target or triple target detection guidelines.

As shown in Figure 1, each experiment requires 60 μl of RNA sample, and each tube (which requires only 10 μl of template) amplifies only one target gene independently and is not mixed for reaction. At the same time, the kit we applied was used to extract viral RNA by magnetic bead method, and 70–80 μl of RNA template could be finally eluted from 300 μl of throat swab each time, which was completely sufficient for the 60 μl of template required by our experiment. This method could complete RNA amplification within 25 min at 39°C (Figure 1). According to the standard curves (Figure 2), our RPA assay improved the detection limit down to 100 copies/ml for M, HA, and PA genes detection (1 copy per reaction) and 200 copies/ml for N gene detection (2 copy per reaction) and 300 copies/ml for E or ORF1ab gene detection (3 copies per reaction), making it more suitable for identifying low‐copy virus infections. Furthermore, the developed RPA assay is extremely specific for SARS‐CoV‐2, H1N1, H3N2, and influenza B. Because we used three target genes to confirm COVID‐19 positivity, and at the same time, we added synchronous testing for influenza A and B, respectively, so that we could quickly identify whether outpatients were suffering from ordinary influenza, infected with SARS‐CoV‐2 or co‐infection. To verify that the established detection technology can be used for the detection of coinfected samples, we added influenza A or B RNA samples (e.g., 104 copies/ml) to the low‐concentration COVID‐19 virus RNA samples (e.g., 104 copies/ml) and performed amplification detection (shown in Online Supporting Information). The results indicated that our method can be used for virus nucleic acid detection of coinfected samples.

**Figure 5:**
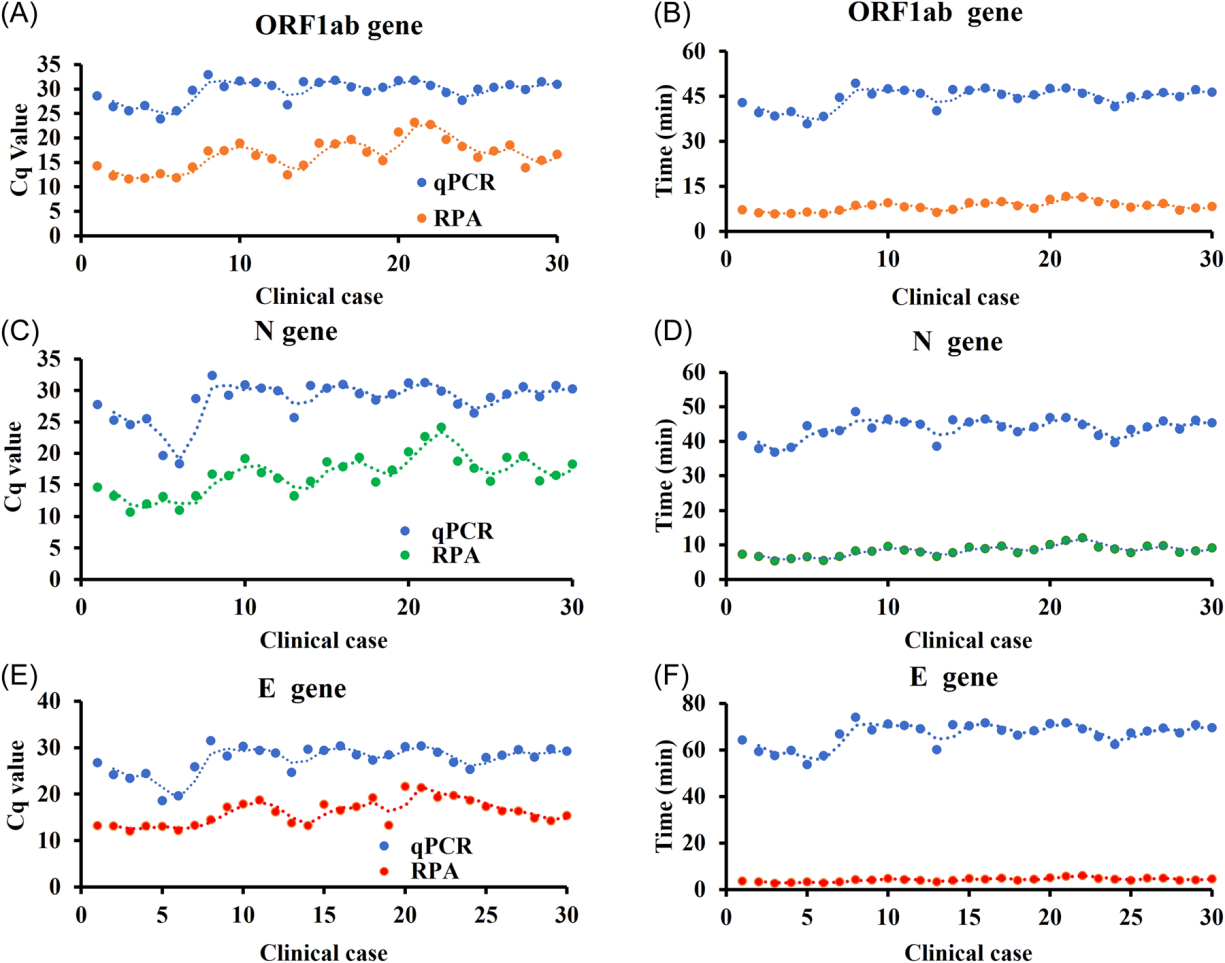
Comparison of amplification results for qPCR and RPA. To compare the amplification effect for RPA and real‐time PCR, 30 samples of COVID‐19 patients were performed the experiment. The Cq value was shown the ORF1ab (A), N (C), and E (E) gene of SARS‐CoV‐2. The time value was shown the ORF1ab (B), N (D), and E (F) gene of SARS‐CoV‐2. These results showed that the detection time of RPA was much faster than that of real‐time PCR. COVID‐19, coronavirus disease 2019; RPA, recombinase polymerase amplification; RT‐PCR, reverse‐transcription polymerase chain reaction.

As shown in Figure 3 and Table 2, RNA samples from COVID‐19 patients (*n* = 70; conformed by RT‐PCR) and healthy donors (*n* = 30) were used for clinical verification, and the accuracy rate for the RPA assay was 100%. The RPA results were 100% consistent with those of RT‐PCR (Table 4), and with the advantage of a shorter assay time (3.39 min vs. 11.17 min) (Figure 5). More importantly, boxplot and ROC analyses showed that the Cq value from COVID‐19 patients was significantly different from that of healthy controls, and the developed RPA assay differentiated COVID‐19 patients from healthy controls with a sensitivity of 100% at a specificity of 90% (Figure 4). The most important thing is that the established detection method can effectively deal with the detection of virus variant strains, and can avoid missing detection (Table 2). It should be noted that, for RPA amplification in this study, fluorescence signals were collected every 30 s; therefore, 30 s was set as the 1 Cq value. In view of the possibility of nonspecific amplification or other background impurity signals for low‐copy samples, we set the cutoff value at 30 Cq for SARS‐CoV‐2, which is the amplification reaction for 15 min. If the amplification time exceeds 15 min, even if there is a weak fluorescent amplification signal, we consider it as a negative result for COVID‐19. Together, the results indicate that the RPA assay holds great promise for detection of SARS‐CoV‐2, H1N1, H3N2, and influenza B, and it could be adapted for POC detection of a broad range of infectious pathogens in resource‐limited settings.

**Table 4:** Comparison of RPA and RT‐PCR amplification results for COVID‐19

		qPCR for COVID‐19		qPCR for influenza A or B	
RPA		Positive	Negative	Positive	Negative
	Positive	70	0	55	0
	Negative	0	30	0	30

*Note*: Sensitivity 100%; Specificity 100%; Accuracy 100%.

Abbreviations: COVID‐19, coronavirus disease 2019; RPA, recombinase polymerase amplification; RT‐PCR, reverse‐transcription polymerase chain reaction.

## 5 CONCLUSION

In summary, we developed an accurate, rapid method for COVID‐19 and influenza diagnosis based on an RPA approach. The assay displayed a sensitivity of 100% for differentiating COVID‐19 and influenza patients from healthy controls when the specificity was at a specificity of 96.67%, 96.67%, and 100% for N, ORF1ab and E gene, and 100%, 100%, and 90% for M, HA, and PA gene, respectively. The assay could potentially be used for screening COVID‐19 and influenza or monitoring infections as an alternative method to RT‐PCR or LAMP in low‐resource settings.

## AUTHOR CONTRIBUTIONS

Hang‐Ping Yao, Zhe Chen, Hang Chen, and Li‐Guo Liang designed the study; Li‐Guo Liang, Miao‐Jin Zhu, Rui He, Dan‐Rong Shi, and Rui Luo writing‐original draft preparation and revised; Supervision, Li‐Guo Liang, Xiang‐Yun Lu, Jia Ji, Wei Lu, Fu‐Ming Liu, Zhi‐Gang Wu, Nan‐Ping Wu; Funding acquisition, Li‐Guo Liang, and Hang‐Ping Yao. All authors reviewed the manuscript and all authors have read and agreed to the published version of the manuscript.

## CONFLICTS OF INTEREST

The authors declare no conflicts of interest.

## Supporting information

Supplementary information.

## Data Availability

The data that support the findings of this study are available from the corresponding author upon reasonable request.
