# Supplementary material for: Development of a multi‐recombinase polymerase amplification assay for rapid identification of COVID‐19, influenza A and B
Source: J Med Virol. 2022 Sep 20:10.1002/jmv.28139. Online ahead of print. doi: 10.1002/jmv.28139 (PMC9538624; doi:10.1002/jmv.28139)
Supplement: Supplementary file 1 — Supplementary information. [file JMV-9999-0-s001.docx]

Supplementary materials

**Development of a multi-recombinase polymerase amplification assay for rapid identification of COVID-19, Influenza A and B**

Li-Guo Liang^a, b, c#^, Miao-jin Zhu^a,b#^, Rui He^d#^, Dan-Rong Shi^a,b^, Rui Luo^a, b^, Jia Ji^a,b^, Lin-Fang Cheng^a,b^, Xiang-Yun Lu^a,b^, Wei Lu^c^, Fu-Ming Liu^a,b^, Zhi-Gang Wu^a,b^, Nan-Ping Wu^a,b^, Hang Chen ^e*^, Zhe Chen^c*^, Hang-Ping Yao^a,b*^

^a^ State Key Laboratory for Diagnosis and Treatment of Infectious Diseases and ^b^ National Clinical Research Center for Infectious Diseases, The First Affiliated Hospital, Zhejiang University School of Medicine, Hangzhou 310003, China

^c^ Center for Clinical Laboratory, The First Affiliated Hospital of Zhejiang Chinese Medical University, 54 Youdian Road, Hangzhou, 310006, China.

^d^ Zhejiang Center for Medical Device Evaluation, Zhejiang Hangzhou, 311121, China

^e^ The Key Laboratory of Biomedical Engineering of Ministry of Education, College of Biomedical Engineering and Instrument Science, Zhejiang University, Hangzhou 310003, China.

In order to verify that the established detection technology can be used for the detection of co-infected samples, we added influenza A or B RNA samples (*e.g.*, 10^4^ copies/mL) to the low-concentration COVID-19 virus RNA samples (*e.g.*, 10^4^ copies/mL) and performed amplification detection. The results are shown in the following table. The method can be used for virus nucleic acid detection of co-infected samples.

Table 1. Simulated detection of co-infected samples

| Results  Groups | Positive | Negative | Coincidence rate (%) |
| --- | --- | --- | --- |
| SARS-CoV-2 +H1N1 | + | - | 100 |
| SARS-CoV-2 +H3N2 | + | - | 100 |
| SARS-CoV-2 +H1N1+H3N2 | + | - | 100 |
